# Temporal patterns in vital sign recording within and across general hospital wards

**DOI:** 10.1016/j.resplu.2022.100247

**Published:** 2022-05-21

**Authors:** Beryl Noë, Alison Bullock, John Frankish, Liam D. Turner

**Affiliations:** a School of Computer Science and Informatics, Cardiff University, UK; b Cardiff Unit for Research and Evaluation in Medical and Dental Education, School of Social Sciences, Cardiff University, UK; c Informatics Directorate, Aneurin Bevan University Health Board, UK

**Keywords:** Vital signs, Early warning score, Observations, Care strategy, CareFlow

## Abstract

**Introduction:**

The use of mobile devices on hospital wards to record patient vital signs and Early Warning Scores provides opportunity for secondary analysis of the data collected. This research investigated how such analysis can contribute to the understanding of the complexities of managing clinical care in hospital environments.

**Methods:**

The influence of ward type and the distribution of patient observation intervals was evaluated in relation to the timing of vital signs observation patterns in data collected from eight adult in-patient wards over a 12-month period. Actual and projected observation times were compared across patients with higher and lower National Early Warning Scores (NEWS).

**Results:**

Both ward type and the distribution of patient observation intervals were significant predictors of temporal observation patterns. Observation patterns showed evidence of grouping of observation recordings. This was, however, not found for observations of patients with higher NEWS scores (3 or more).

**Conclusions:**

Secondary analysis of vital signs observation data can reveal insights into how ward operate. The patterns of observation recordings within a ward are a reflection of ward type and the distribution of patient observation intervals. The grouping of observation recordings of patients with low NEWS (<3) result in late or early observations to fit activity peaks characteristic of the ward culture.

## Introduction

Routine observations of patient vital signs are scheduled to conform with clinical policies that determine the recording interval based on a patient’s individual health needs.^1^ However, this process is affected by a variety of factors including staffing levels and available skills and experience.2., 3. Moreover, as the process creates a set of individual observation schedules, it has been argued that conformance is further impacted by the complexity of competing clinical priorities.^4^ While conformance challenges have been demonstrated in specific case studies,5., 6., 7.,they have not been widely explored in a longitudinal context across different types of wards and hospital sites.

The introduction of mobile devices on wards to record patient vital signs enables a holistic secondary analysis of the data collected. The aim of this research is to examine how such analysis can provide an extended and complementary data-driven insight into the complexities of managing clinical care in in-patient environments.

## Methods

### Study design

We conducted an analysis of an anonymised dataset of patient observation and vital sign recordings over 12-months, across multiple wards and hospital sites. We extracted aggregated temporal patterns of when and how observations are conducted. From this, we exposed homogenous and heterogenous behaviours across wards that are indicative of the complexities of managing clinical priorities within wards and discovered that common heuristics are used to balance competing policies, individual patient care needs (including restful sleep), and other events in the day such as shift changes.

### Study setting and period

Routine vital sign recording data was obtained from eight wards located at two Aneurin Bevan University Health Board (ABUHB) hospitals in Wales for the uninterrupted period of one year. All data stems from observations recorded in 2019 before the start of the COVID-19 pandemic in the UK in February 2020.^8^

The study was approved by a Research Ethics Committee at Cardiff University (reference number: SREC/3842). Additionally, Aneurin Bevan University Health Board's Research and Development Department gave a favourable opinion of the service evaluation underpinning the study (reference number: SA/1148/20).

### Data overview

Data were captured from two different sites to encompass variety. Three types of general wards were included: medical, surgical, and rehabilitation wards. For each hospital, data were obtained for two pairs of wards of different types. Data for medical wards were acquired for both sites, while data from surgery and rehabilitation wards were collected respectively from hospital 1 and 2 (see Table 1).

**Table 1:** Summary of ward characteristics for each of the eight study wards.

Hospital and ward identifier	H1S1	H1S2	H1M1	H1M2	H2M1	H2M2	H2R1	H2R2
ward type	surgery	surgery	medical	medical	medical	medical	rehab	rehab
number of staff IDs	278	257	204	139	147	117	133	162
number of patient IDs	1362	1607	907	1424	1177	404	359	315
number of beds	32	32	32	30	18	30	30	30
number of observations	38,376	36,122	37,705	36,794	24,663	23,755	24,248	22,468

H = Hospital, S = Surgery ward, M = Medical ward, R = Rehabilitation ward.

Staff and patient data were anonymised. Staff IDs are indicative of the accounts associated with the input of vital sign observation data and can be shared (e.g., by agency staff). The number of staff IDs given in Table 1 therefore does not reflect the exact number of staff in charge of patient observations. Every patient admitted to the hospital has an assigned ID which is tied to their observations for the entirety of their hospitalisation until discharge. The number of patient IDs therefore accurately reflects the number of patients staff interact with on a daily basis. A total of 244,131 observations were collected across all study wards.

### Observation recordings

The use of mobile devices equipped with the electronic software CareFlow (formerly VitalPAC)^9^ has been implemented to record patient vitals for a year or more in both study hospitals. During each bedside observation, the software requests specific vital signs to be measured and recorded, such as heart rate, respiratory rate, and oxygen saturation. On the basis of these measures, the software then automatically calculates the National Early Warning Score (NEWS) for the examined patient.

NEWS is a tool allowing the rapid assessment of the degree of illness of a patient by assigning a single score summarising how far from the normal range the recorded vital signs are. Based on the hospital’s escalation policy (here, ABUHB’s Deteriorating Patient Policy^1^), this score, combined with the history of NEWS scores then determines the observation frequency, i.e. the time interval until the patient should be seen again. The observation frequency is typically shorter for patients with higher NEWS scores who are considered more unwell and for patients with a recent deterioration in NEWS score. Both the NEWS score and the observation frequency are displayed to the staff once the full vital sign set has been submitted.

Additionally, the following measures are also recorded in the database: the time at which the observation is started and submitted, the ID of the staff submitting the observation, the ID of the examined patient, and the location of the patient (hospital, ward, bay, and bed IDs).

### Definition of ‘on time’, ‘late’, and ‘missed’ observations

To the best of our knowledge, no formal definition of ‘late’ and ‘missed’ observations exist within the Health Board's policies. In the literature, little research has been carried out focusing on the conformance to observation intervals and definitions vary4., 10., 11.. To encompass variability, we have opted for the distinction of ‘late’ observations in two categories: ‘A’ (which may also be considered ‘on time’ in other studies4., 10.) and ‘B’.

An observation is qualified as ‘on time’ when it happens within the time-to-next-observation (TTNO) displayed by CareFlow. Observations are defined as ‘late’ when they occur after the TTNO has passed, but are not overdue by more than 100%. Within this ‘late’ appellation, we make the distinction between two levels of lateness: ‘late A’, which encompasses observations that occur between 0% and 33% of the TTNO and ‘late B’, which comprises the observations that occur between 33% and 100% of the TTNO.

The number of ‘missed’ observations is calculated when the time between two observations exceeds the TTNO by more than 100%. It is determined by how many observations should have occurred if the observation schedule had been followed. There may be valid reasons for when observations do not occur, e.g. a ward change for a patient, but the number of missed observations is capped at a maximum of two sequential missed observations.

These definitions led to the observations counts shown in Table 2, where an overview of the number of ‘on time’, ‘late’, and ‘missed’ observations can be seen. The total number of observations is also given in this table, which excludes ‘missed’ observations as these did not occur and were calculated separately.

**Table 2:** Summary of observation data for each of the eight study wards.

ward	H1S1	H1S2	H1M1	H1M2	H2M1	H2M2	H2R1	H2R2
on time	23,287 (61%)	22,116 (61%)	20,276 (54%)	20,710 (56%)	18,568 (75%)	13,147 (55%)	12,648 (52%)	11,160 (50%)
late A	8224 (21%)	7423 (21%)	9938 (26%)	9467 (26%)	2906 (12%)	8866 (37%)	6267 (26%)	6844 (30%)
late B	6865 (18%)	6583 (18%)	7491 (20%)	6617 (18%)	3189 (13%)	1742 (7%)	5333 (22%)	4464 (20%)
*Total observations*	*38,376 (100%)*	*36,122 (100%)*	*37,705 (100%)*	*36,794 (100%)*	*24,663 (100%)*	*23,755 (100%)*	*24,248 (100%)*	*22,468 (100%)*
missed	3060	2348	3586	3303	2197	1234	2491	2009

### Analysis

Late and missed observations are indicative of the challenges surrounding conformance to hospital policies dictating TTNO based on patient condition. The aim of this research is to examine how the analysis of bedside vital sign observations collected through mobile devices can contribute to a better understanding of the complexities of clinical care management in hospital wards.

The number of total observations was calculated per hour of day and patient EWS for every ward and classified as ‘on time’, ‘late A’, or ‘late B’. The number of ‘missed’ observations were computed likewise.

Additionally, by calculating when observations *should* have happened according to the TTNO determined at the previous observation, we were able to compute the distribution of “projected” observations. Further, the proportions of different TTNOs can be calculated for each ward, resulting in a ward-specific “TTNO distribution”.

The aggregated set of observations in a day can be represented as a distribution of the number of observations per hour of day. This is referred to as the “temporal observation patterns” in the analysis.

### Statistical analysis

#### Observation activity patterns

Descriptive statistics were computed using counts and percentages.

#### Variances between actual and projected observation distributions

The difference in variances between the distribution of “actual” observations and “projected” observations was evaluated using Levene’s test for each ward. The number of observations were aggregated per hour of day. Observations for which a projected observation time could not be computed were disregarded.

#### Prediction of temporal observation patterns

Negative binomial generalised linear models were used to predict the number of observations per hour of day based on the categorisation of wards based on TTNO distribution and ward type respectively. TTNO distribution type was determined based on the distribution of TTNOs for each ward. The first group consisted of wards with a majority of 6 h-, 8 h- and 1 h-TTNOs: H1S1, H1S2, H1M1, H1M2, and H2M1. The second group on the other hand was mainly composed of observations undertaken on a 12 h-basis: H2M2, H2R1, and H2R2.

For both models, the number of staff that had recorded observations for the same day within a ward was introduced as a confounding factor in the regression to take into account different wards sizes. Additionally, the number of observations recorded in the previous hour and the month in which the observations were recorded were also taken into account to correct respectively for the effects of auto-correlation and seasonality. The day of the month was initially also considered, but as it was not a significant predictor, it was removed in the final models. These models were then compared using a Vuong test.

## Results

### Activity peaks in patterns of observations

When aggregating the number of observations throughout the year per hour of day, activity peaks were observed for each of the wards.

While the number of peaks varied per ward (2 to 4), all wards displayed a clear morning and evening peak. For instance, 38.93% of the observations in the ward with four peaks (H2M2) occurred in a span of 4 hours between 05:00 and 07:00 in the morning and 19:00 and 21:00 in the evening. This percentage ranged from 45.87% to 50.18% for wards with 3 peaks (all H1 wards and H2M1) when considering the same timeframes. For wards with two activity peaks, a clear distinction is found in ward types. In the medical ward (H2M2), 73.7% of all daily observations occurred within 4 hours (between 05:00 and 07:00 in the morning and 15:00 and 17:00 in the afternoon). In contrast, the observation peaks in the rehabilitation wards were more spread out: 77.62% (H2R1) and 80.55% (H2R2) of all observations occurred within 7 hours (07:00 to 12:00 in the morning and 19:00 to 21:00 in the evening).

### Downtime in patterns of observations

Across all wards a consistent downtime was observed in a time span of 5 hours in the early morning (between 00:00 and 5:00), where altogether only 4.07% of all observations occurred. This percentage varied per ward between 1.66% and 8.28%.

### Variances between actual and projected observation distributions

**Fig. 1:**
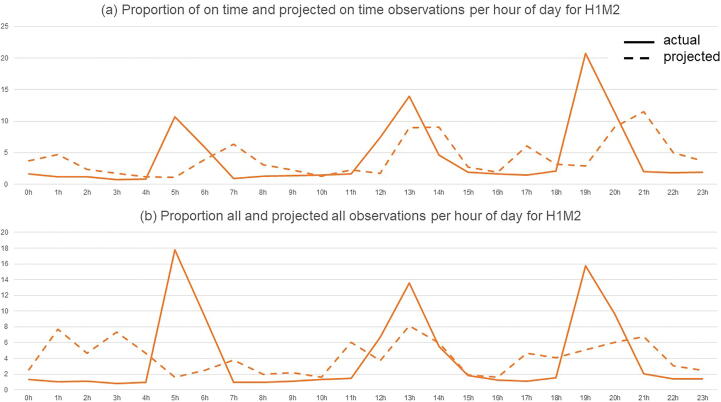
Proportion of actual and projected observations for an example ward, considering only (a) on-time observations and (b) all observations.

For all but three wards (H1S2, H2M2, and H2R2, see Table 3), variances were found to be statistically different (p <.05), indicating the presence of higher activity peaks in actual observation patterns in comparison to projected observation patterns for most wards. Fig. 1 shows the proportion of actual and projected observations for one ward. Peaks can be observed in the projected observation line for late observations preceding actual observation peaks and for on-time observations following the actual observation peak.

**Table 3:** Summary of results of equality of variances tests.

	variance_actual_	variance_projected_	F	p
**All NEWS**				
H1S1	1040630	284661.3	4.6405	0.0365
H1S2	1656484	367187.1	3.6225	n.s
H1M1	971204.4	245406.1	6.1231	0.01709
H1M2	1080556	314996.9	4.6406	0.0365
H2M1	646009.2	204791.4	8.2685	0.006092
H2M2	1114236	1314817	0.0318	n.s
H2R1	319776.7	131884.1	6.3091	0.01558
H2R2	343349.9	187073.9	2.0475	n.s.

**NEWS ≥ 3**				
H1S1	26393.26	27,399	0.0276	n.s.
H1S2	47188.75	48710.49	0.0043	n.s.
H1M1	60139.91	44537.91	0.1817	n.s.
H1M2	125704.1	54923.22	2.4853	n.s.
H2M1	16710.41	15965.88	0.0161	n.s.
H2M2	13840.87	15327.13	0.113	n.s.
H2R1	2604.607	2898.259	0.6975	n.s.
H2R2	3068.868	2689.824	0.0232	n.s.

**NEWS ≥ 6**				
H1S1	325.2971	456.9493	1.025	n.s.
H1S2	332.3025	479.0851	0.276	n.s.
H1M1	1368.824	780.8243	0.9328	n.s.
H1M2	2128.81	1139.245	1.0637	n.s.
H2M1	385.2591	334.3895	1.0479	n.s.
H2M2	174.8098	175.4185	0.5455	n.s.
H2R1	33.71014	40.75362	0.8587	n.s.
H2R2	33.91123	31.04167	0.0275	n.s.

Levene’s tests were repeated on data taking only into account observations of patients with previously higher NEWS scores. No statistically significant differences in variances between the actual and projected observation patterns were found for both sets of analyses considering observations with NEWS scores equal or higher than 3 and NEWS scores equal or higher than 6 (see Table 3).

### TTNO distribution and ward type predicts temporal observation patterns

**Fig. 2:**
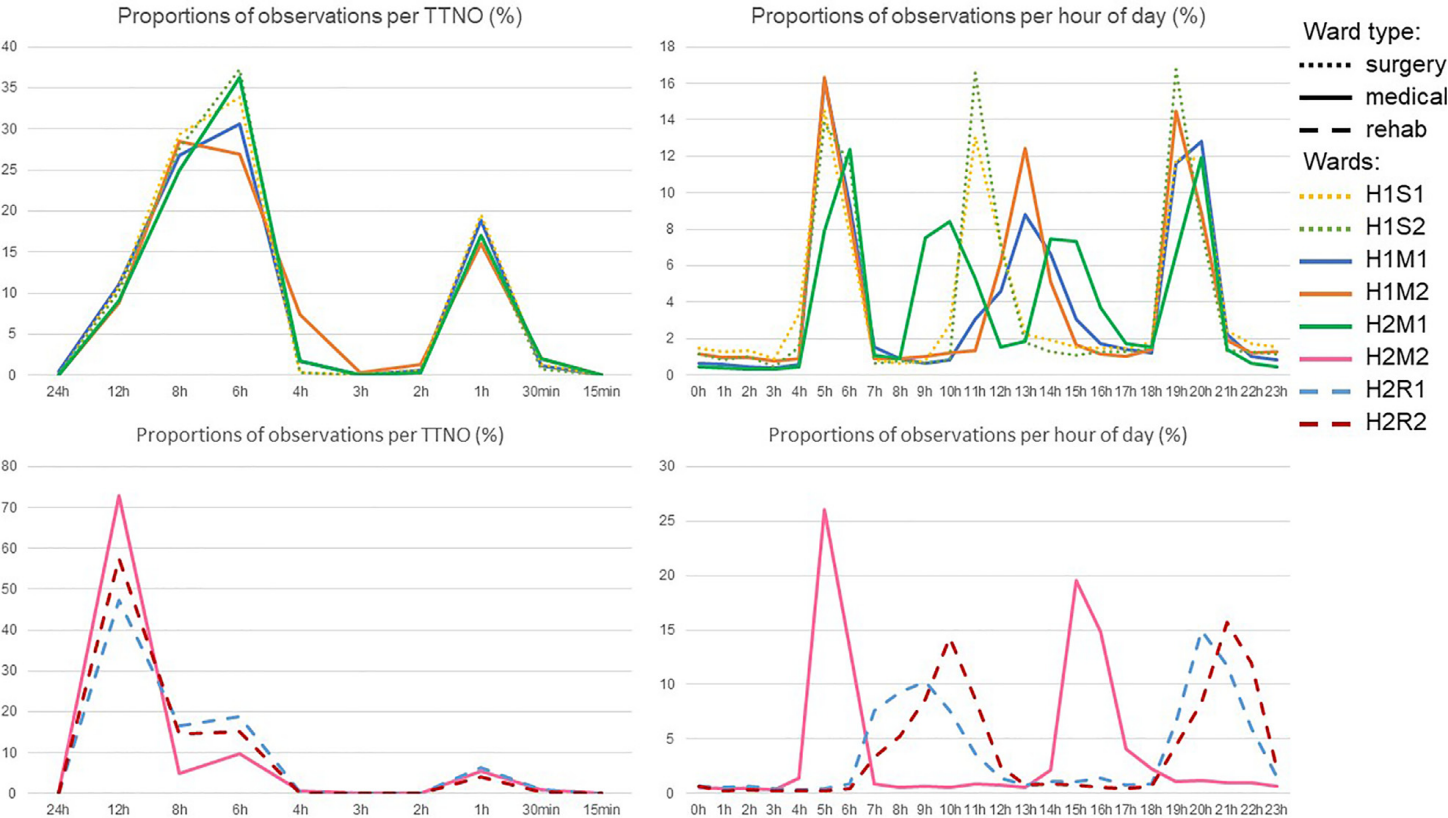
Proportion of observations per TTNO and per hour of day for all wards.

The two different categories of TTNO distribution can be seen in Fig. 2, which shows both the distribution or TTNO intervals and the proportion of observations throughout the day.

Results showed that the categorisation by TTNO distribution (p <.001) was a significant predictor for the number of observations per hour of day, however ward type was only a significant predictor when medical and surgery ward types were combined (p <.001) (see Table 4).

**Table 4:** Summary of final Negative binomial generalised linear models.

	Estimate	Std. Error	z	p
**Ward type model**				
Intercept	0.2373827	0.0324214	7.322	<0.001
**Ward type**	**−0.1472149**	**0.016358**	**−9**	**<0.001**
Hour of day	0.008506	0.0009272	9.174	<0.001
Number of staff	0.0643933	0.0021333	30.184	<0.001
Number observations previous hour	0.0532486	0.0011088	48.023	<0.001
Month	−0.0087775	0.0018539	−4.735	<0.001

**TTNO distribution type model**				
Intercept	0.1456514	0.0291202	5.002	<0.001
**TTNO distribution type**	**0.2029244**	**0.0147665**	**13.742**	**<0.001**
Hour of day	0.00799	0.0009264	8.624	<0.001
Nbr of staff	0.0586057	0.0021563	27.178	<0.001
Nbr observations previous hour	0.0531158	0.001109	47.896	<0.001
Month	−0.0088843	0.0018523	−4.796	<0.001

When compared using the Vuong test, the model based on the TTNO distribution (AIC = 307,622) performed significantly better (p <.001) than the model based on ward type (AIC = 307,685).

## Discussion

### Observations

Although the Deteriorating Patient Policy^1^ is common to all of the Health Board’s sites and wards, varying proportions of on-time, late, and missed observations were noted across the study wards (see Table 2). Similarities and differences in temporal observation patterns were further revealed, providing insight into the management of vital sign recordings.

Activity peaks and downtimes were observed across the whole sample. Notable similarities found in all wards were the occurrence of at least 2 peaks, one of which happened in the morning and one of which occurred in the late afternoon (H2M2) or evening. Another phenomenon that was observed consistently across all wards was downtime occurring between midnight and 05:00. This downtime is the result of “restful sleep”, a common practice in in-patient care during which sleep is prioritised over waking up patients for which frequent monitoring is less critical.12., 13., 14., 15.

Further examination of temporal observation patterns suggests that observations are performed in rounds that occur two to four times per day with downtime in between. Results from the Levene’s tests comparing projected with actual observation patterns and the finding of significant variances in activity for the majority of the wards, suggest that observations are not spread out evenly throughout the day. We note instead that they are grouped together creating activity peaks and downtimes responsible for a higher variance of observation patterns. Late observations seem not only to be pushed to the next activity peak, but on-time observations seem to be pulled forward as well (see Fig. 1). The grouping of observations occurs to a lesser extent for patients in a more critical condition however. Indeed, no significant differences in variances were found for actual and projected observation patterns of patients scoring 3 and above on the NEWS scale. This means that a different observation management approach is used for patients who are more at risk, which may be influenced by nurse staffing levels^2^ and recommendations for risk stratification of patients to ensure fewer overnight disruptions.^16^

When surgery and medical ward types were combined, ward type was found to be a significant predictor of temporal observation patterns. While certain characteristics could be found within the observation patterns of each ward, differences per ward type could be observed, for instance, in the timing of the morning peak: for surgery and medical wards, this peak occurred before the start of the morning shift at 07:00, while it was situated thereafter for rehabilitation wards. Activity peaks were also spread over a longer time periods in rehabilitation wards. To illustrate, the morning activity peak spanned 5 hours (from 07:00 to 12:00) in rehabilitation wards encompassing 38% to 40% of all daily observations, whereas in surgical and medical wards morning activity peaks spanned 2 hours (from 05:00 to 07:00), encompassing 20% to 40% of all daily observations. These results are indicative of different choices in observation management for different ward types and suggest the influence of ward culture on temporal observation patterns.

Proportional distribution of patients’ TTNOs too were determined to be significant predictors for temporal observation patterns. Indeed, we observe that wards with a majority of patients being observed on a 12-hour schedule have two activity peaks situated about 10 to 12 hours apart, while wards with a majority of 6-to-8-hour-TTNOs have three to four activity peaks situated 3 to 7 hours apart within the daytime. We note that this is in accordance to NICE guidance,^17^ which prescribes a minimum of 12-hourly observations. While these results are unsurprising, it is worth noting that to the best of our knowledge, this is the first time ward-level data have been examined with this categorisation in mind. Such analysis enables meaningful comparisons to be drawn between ward types and different hospitals. For example, ward H2M2 has, unlike other medical wards in our sample, a majority of 12-hour-TTNO observations. While the pattern of observations per time of day in H2M2 show overlap with other medical wards, discrepancies are noted too. Indeed, ward H2M2′s first activity peak coincides with other medical wards’ morning peak and its second peak falls between other medical wards’ afternoon and evening peaks. We note that in comparison to the rehabilitation wards that also have a majority of 12-hour-TTNO observations, the activity peaks are pulled forward however, resulting in an early waking of patients at 05:00 and 06:00. This could be avoided if the second activity peak was pushed further back as seems to happen in the rehabilitation wards.

### Clinical implications

We note differences in patterns of vital signs observations per wards. Particularly, we recognise that the activity peaks for patients with more stable observations represent deliberate clinical rounding to ‘do the observations’ and that the patterns of peaks for some wards is not evenly distributed with long gaps between standard observations. Moreover, the practice of doing vitals during periods that should be set aside for restful sleep where possible is observed for most of the study wards. Based on these findings, we suggest:

(1) For hospital wards with a high number of short observation frequencies, typically hosting patients with higher NEWS scores, if observations are spread over 4 activity peaks rather than 3 this could result in fewer late and missed observations.
(2) The schedule of observations for patients with low NEWS scores (or where it is clinically safe to do so) should avoid disturbance of their restful sleep.

More generally, ward management could be supported by providing an overview of observation patterns and patient observation needs. This information equips clinicians with the insight to evaluate their ward practices with a view to ensuring distribution of standard observations rounds evenly in the interests of patient safety to eliminate the possibilities of long gaps between observations occurring. These data could be therefore be used both for the review of ward practices and for the support decision making for patient management. Monitoring the patient cohort at a ward level could especially be beneficial when looked at per different levels of NEWS scores. Similar to the progress observed by the implementation of electronic patient record system,^18^ we expect that providing further relevant patient data overviews will improve the monitoring of patients and accelerate the recognition and response to clinical deterioration, which is key in reducing mortality.^19^

## Limitations and future work

This study focused on a relatively small number of wards, which limits the conclusions that can be drawn from the results. These results however indicate that the analysis of observation patterns can yield valuable information, which can be used to support decision making for patient management. Future research should aim to broaden the current work by increasing the number of wards and sites examined. A further limitation is that we did not analyse the data by patient condition; future research could expand on patient health data, perhaps focusing especially on sepsis given the gravity of the consequences.

## Conclusions

In this paper we have demonstrated how secondary analysis of observation data can reveal insights into patterns of ward operation. Through the analysis of daily and hourly aggregated data, we identified activity peaks that occur throughout the day for each ward. While wards had differences in the number of peaks, they commonly perform observations one after another. As a result, observations scheduled before a typical activity peak can often be late, and observations scheduled to happen later can be completed early. However, these patterns are not seen for high NEWS scores; these patients are at greater risk and individualised care is provided. Additionally, we found that the timing and number of activity peaks was not random, and the observation patterns were associated with ward type and the TTNO distribution. Overall, the results show that analysing observation behaviour across a ward, rather than on a per-patient basis, can provide additional contextual information about why observations may be late or early. This also highlights the complexities of managing patient care in the context of both formal policies and individual patient needs.

## Funding

This research was funded by the Nevill Hall Hospital Thrombosis and General Research Fund charity. This research was also supported by The Centre for Artificial Intelligence, Robotics and Human-Machine Systems, Cardiff University.

## CRediT authorship contribution statement

**Beryl Noë:** Conceptualization, Data curation, Formal analysis, Funding acquisition, Investigation, Methodology, Software, Visualization, Writing – original draft, Writing – review & editing. **Alison Bullock:** Conceptualization, Funding acquisition, Investigation, Methodology, Project administration, Supervision, Writing – review & editing. **John Frankish:** Conceptualization, Data curation, Funding acquisition, Resources, Writing – review & editing. **Liam D. Turner:** Conceptualization, Funding acquisition, Investigation, Methodology, Project administration, Software, Supervision, Writing – original draft, Writing – review & editing.

## Declaration of Competing Interest

The authors declare that they have no known competing financial interests or personal relationships that could have appeared to influence the work reported in this paper.
